# Soil bacterial community composition of different tropical land use systems in Jambi province, Indonesia

**DOI:** 10.1128/mra.01018-24

**Published:** 2025-04-14

**Authors:** Dirk Berkelmann, Lena Luisa Suhl, Dominik Schneider, Anja Meryandini, Rolf Daniel

**Affiliations:** 1 Genomic and Applied Microbiology and Göttingen Genomics Laboratory, Institute of Microbiology and Genetics, Georg-August-University 214613 , Göttingen, Germany; 2 Department of Biology, Faculty of Mathematics and Natural Sciences IPB, Bogor Agricultural University, Bogor, Indonesia; DOE Joint Genome Institute, Berkeley, California, USA

**Keywords:** oil palm, soil bacterial communities, rainforest conversion, rubber, active soil bacterial community, V3-V4 region

## Abstract

We sequenced the V3-V4 region of 16S rRNA genes and transcripts to assess entire (DNA) and active (RNA) soil bacterial communities in four different Indonesian land use systems (jungle rubber, rubber, oil palm, and rainforest). *Acidobacteriota* and *Planctomycetes* were dominant, with higher relative abundances at active community level.

## ANNOUNCEMENT

This study was part of the EFForTS project (Ecological and socioeconomic Functions of tropical lowland rainForest Transformation Systems) in Jambi province, Indonesia (1). We compared secondary lowland rainforest with converted land use systems: jungle rubber (*Hevea brasiliensis* planted in forest), rubber (*Hevea brasiliensis* monocultures), and oil palm (*Elaeis guineensis* monocultures) to investigate the effects of land use intensity on bacterial communities (1). For each land system, four coreplots consisting of three subplots were analyzed (see Table 1). Topsoil (upper 7 cm) was sampled in May 2017 of each subplot with a soil corer (5 cm diameter). Three cores were taken per subplot with 1 m distance, pooled, and supplemented with RNAprotect Bacteria Reagent (Qiagen, Hilden, Germany) to prevent RNA degradation. Samples were transported in cooling boxes and stored at −80 °C.

**TABLE 1:** Sample list including land use type, plot coordinates, as well as sequencing and processing numbers

Sample ID	Coreplot	Land use	Template	Latitude	Longitude	Raw reads	Reads after filtering
BF1a	BF1	Rainforest	DNA	−1.995139	102.75225	72,460	42,985
BF1a	BF1	Rainforest	RNA	−1.995139	102.75225	75,469	40,009
BF1b	BF1	Rainforest	DNA	−1.995139	102.75225	71,555	51,323
BF1b	BF1	Rainforest	RNA	−1.995139	102.75225	84,791	50,037
BF1c	BF1	Rainforest	DNA	−1.995139	102.75225	79,137	54,342
BF1c	BF1	Rainforest	RNA	−1.995139	102.75225	96,712	55,867
BF2a	BF2	Rainforest	DNA	−1.981972	102.75075	120,399	84,420
BF2a	BF2	Rainforest	RNA	−1.981972	102.75075	86,970	49,824
BF2b	BF2	Rainforest	DNA	−1.981972	102.75075	75,951	55,963
BF2b	BF2	Rainforest	RNA	−1.981972	102.75075	80,546	50,171
BF2c	BF2	Rainforest	DNA	−1.981972	102.75075	139,308	87,295
BF2c	BF2	Rainforest	RNA	−1.981972	102.75075	118,343	54,372
BF3a	BF3	Rainforest	DNA	−1.943290187	102.5811431	65,660	50,954
BF3a	BF3	Rainforest	RNA	−1.943290187	102.5811431	74,514	42,842
BF3b	BF3	Rainforest	DNA	−1.943290187	102.5811431	58,381	42,590
BF3b	BF3	Rainforest	RNA	−1.943290187	102.5811431	75,000	48,572
BF3c	BF3	Rainforest	DNA	−1.943290187	102.5811431	99,595	70,119
BF3c	BF3	Rainforest	RNA	−1.943290187	102.5811431	188,839	115,242
BF4a	BF4	Rainforest	DNA	−1.941875322	102.5807841	63,642	47,230
BF4a	BF4	Rainforest	RNA	−1.941875322	102.5807841	76,559	47,720
BF4b	BF4	Rainforest	DNA	−1.941875322	102.5807841	23,152	17,349
BF4b	BF4	Rainforest	RNA	−1.941875322	102.5807841	106,245	72,080
BF4c	BF4	Rainforest	DNA	−1.941875322	102.5807841	56,969	41,477
BF4c	BF4	Rainforest	RNA	−1.941875322	102.5807841	53,770	41,978
BJ2a	BJ2	Jungle rubber	DNA	−2.030269364	102.7709138	55,707	42,966
BJ2a	BJ2	Jungle rubber	RNA	−2.030269364	102.7709138	89,965	52,257
BJ2b	BJ2	Jungle rubber	DNA	−2.030269364	102.7709138	69,147	53,303
BJ2b	BJ2	Jungle rubber	RNA	−2.030269364	102.7709138	98,371	58,782
BJ2c	BJ2	Jungle rubber	DNA	−2.030269364	102.7709138	95,766	59,738
BJ2c	BJ2	Jungle rubber	RNA	−2.030269364	102.7709138	107,034	61,441
BJ3a	BJ3	Jungle rubber	DNA	−2.063536048	102.8007258	68,003	52,398
BJ3a	BJ3	Jungle rubber	RNA	−2.063536048	102.8007258	100,693	59,262
BJ3b	BJ3	Jungle rubber	DNA	−2.063536048	102.8007258	71,710	55,704
BJ3b	BJ3	Jungle rubber	RNA	−2.063536048	102.8007258	89,730	57,100
BJ3c	BJ3	Jungle rubber	DNA	−2.063536048	102.8007258	81,453	48,625
BJ3c	BJ3	Jungle rubber	RNA	−2.063536048	102.8007258	80,324	46,410
BJ4a	BJ4	Jungle rubber	DNA	−2.015788788	102.7532639	64,401	49,312
BJ4a	BJ4	Jungle rubber	RNA	−2.015788788	102.7532639	76,625	44,171
BJ4b	BJ4	Jungle rubber	DNA	−2.015788788	102.7532639	84,873	59,231
BJ4b	BJ4	Jungle rubber	RNA	−2.015788788	102.7532639	121,380	70,148
BJ4c	BJ4	Jungle rubber	DNA	−2.015788788	102.7532639	59,093	41,806
BJ4c	BJ4	Jungle rubber	RNA	−2.015788788	102.7532639	77,735	45,053
BJ5a	BJ5	Jungle rubber	DNA	−2.143267561	102.8512447	67,740	59,203
BJ5a	BJ5	Jungle rubber	RNA	−2.143267561	102.8512447	71,384	45,057
BJ5b	BJ5	Jungle rubber	DNA	−2.143267561	102.8512447	60,472	50,691
BJ5b	BJ5	Jungle rubber	RNA	−2.143267561	102.8512447	65,595	41,153
BJ5c	BJ5	Jungle rubber	DNA	−2.143267561	102.8512447	73,438	58,613
BJ5c	BJ5	Jungle rubber	RNA	−2.143267561	102.8512447	97,069	65,728
BO2a	BO2	Oil palm	DNA	−2.075623842	102.7924752	71,155	53,279
BO2a	BO2	Oil palm	RNA	−2.075623842	102.7924752	69,890	41,257
BO2b	BO2	Oil palm	DNA	−2.075623842	102.7924752	88,447	66,061
BO2b	BO2	Oil palm	RNA	−2.075623842	102.7924752	108,158	59,494
BO2c	BO2	Oil palm	DNA	−2.075623842	102.7924752	67,019	53,625
BO2c	BO2	Oil palm	RNA	−2.075623842	102.7924752	78,751	46,753
BO3a	BO3	Oil palm	DNA	−2.071417635	102.7921114	58,883	42,847
BO3a	BO3	Oil palm	RNA	−2.071417635	102.7921114	68,809	38,456
BO3b	BO3	Oil palm	DNA	−2.071417635	102.7921114	96,853	73,397
BO3b	BO3	Oil palm	RNA	−2.071417635	102.7921114	137,474	80,580
BO3c	BO3	Oil palm	DNA	−2.071417635	102.7921114	82,126	59,673
BO3c	BO3	Oil palm	RNA	−2.071417635	102.7921114	92,160	51,078
BO4a	BO4	Oil palm	DNA	−2.050463129	102.753405	77,448	58,281
BO4a	BO4	Oil palm	RNA	−2.050463129	102.753405	90,149	55,333
BO4b	BO4	Oil palm	DNA	−2.050463129	102.753405	90,196	64,962
BO4b	BO4	Oil palm	RNA	−2.050463129	102.753405	85,390	52,207
BO4c	BO4	Oil palm	DNA	−2.050463129	102.753405	84,390	62,524
BO4c	BO4	Oil palm	RNA	−2.050463129	102.753405	75,910	51,492
BO5a	BO5	Oil palm	DNA	−2.114060484	102.7951525	112,827	83,381
BO5a	BO5	Oil palm	RNA	−2.114060484	102.7951525	121,022	70,460
BO5b	BO5	Oil palm	DNA	−2.114060484	102.7951525	80,250	58,359
BO5b	BO5	Oil palm	RNA	−2.114060484	102.7951525	92,115	54,382
BO5c	BO5	Oil palm	DNA	−2.114060484	102.7951525	73,055	58,169
BO5c	BO5	Oil palm	RNA	−2.114060484	102.7951525	76,941	47,331
BR1a	BR1	Rubber	DNA	−2.092406675	102.8030459	78,588	59,470
BR1a	BR1	Rubber	RNA	−2.092406675	102.8030459	72,011	40,498
BR1b	BR1	Rubber	DNA	−2.092406675	102.8030459	65,426	46,911
BR1b	BR1	Rubber	RNA	−2.092406675	102.8030459	67,059	38,594
BR1c	BR1	Rubber	DNA	−2.092406675	102.8030459	69,839	57,343
BR1c	BR1	Rubber	RNA	−2.092406675	102.8030459	106,666	59,176
BR2a	BR2	Rubber	DNA	−2.085339222	102.7895367	73,445	55,302
BR2a	BR2	Rubber	RNA	−2.085339222	102.7895367	86,602	51,291
BR2b	BR2	Rubber	DNA	−2.085339222	102.7895367	57,349	46,712
BR2b	BR2	Rubber	RNA	−2.085339222	102.7895367	101,463	58,151
BR2c	BR2	Rubber	DNA	−2.085339222	102.7895367	54,027	42,330
BR2c	BR2	Rubber	RNA	−2.085339222	102.7895367	84,289	50,320
BR3a	BR3	Rubber	DNA	−2.096007206	102.7832653	60,779	47,693
BR3a	BR3	Rubber	RNA	−2.096007206	102.7832653	82,458	53,562
BR3b	BR3	Rubber	DNA	−2.096007206	102.7832653	107,997	87,209
BR3b	BR3	Rubber	RNA	−2.096007206	102.7832653	104,501	70,242
BR3c	BR3	Rubber	DNA	−2.096007206	102.7832653	62,593	48,545
BR3c	BR3	Rubber	RNA	−2.096007206	102.7832653	62,761	40,109
BR4a	BR4	Rubber	DNA	−2.077258984	102.7731989	49,518	35,553
BR4a	BR4	Rubber	RNA	−2.077258984	102.7731989	56,859	32,052
BR4b	BR4	Rubber	DNA	−2.077258984	102.7731989	75,304	59,097
BR4b	BR4	Rubber	RNA	−2.077258984	102.7731989	100,678	60,840
BR4c	BR4	Rubber	DNA	−2.077258984	102.7731989	84,996	48,241
BR4c	BR4	Rubber	RNA	−2.077258984	102.7731989	105,969	61,665

**Fig 1:**
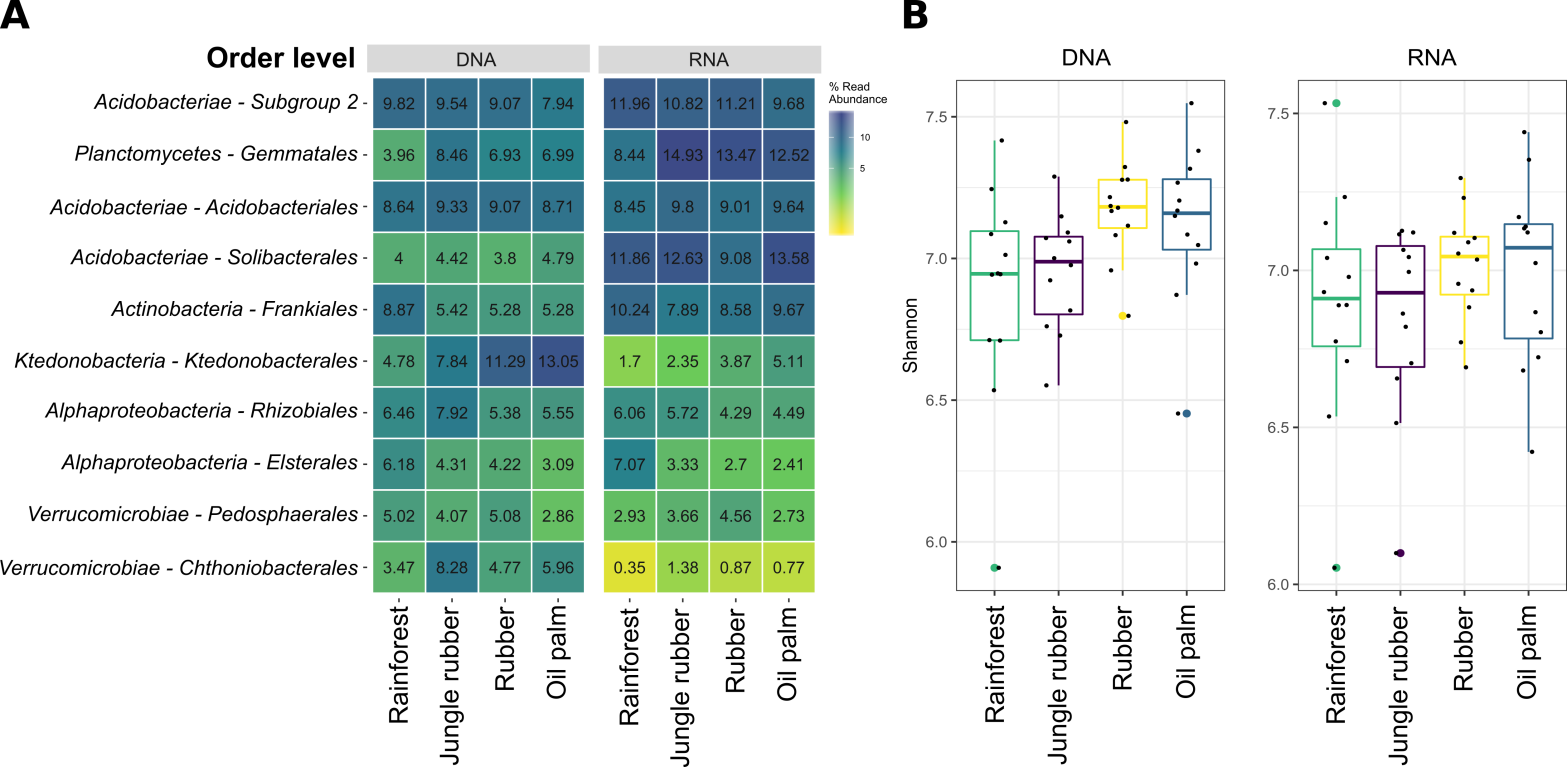
Taxonomic composition and beta diversity at entire and active bacterial community level. (**A**) The ten most abundant bacterial orders from the entire and active community of the respective land use system as relative abundances. (**B**) Beta diversity displayed as Shannon diversity index for the entire and active community of the respective land use system. Diversity indices were calculated at ASV level with the ampvis2 package, which utilizes the vegan function “diversity.”

RNAprotect was removed after sample thawing on ice by centrifugation for 20 min at 804 *g* and 4°C. Nucleic acid extractions were done by using 1 g of soil with the Qiagen RNeasy PowerSoil Total RNA kit and the RNeasy PowerSoil DNA Elution kit (Qiagen) as recommended by the manufacturer, except using 50 µl elution buffer for elution of RNA. DNA was removed from RNA extracts with the TurboDNAfree kit (Applied Biosystems, Darmstadt, Germany) with an additional DNase digestion cycle of 15 min at 37 °C. RNA was purified with the RNeasy MiniElute Cleanup kit (Qiagen), and DNA removal was controlled by PCR targeting the 16S rRNA gene as described below. cDNA synthesis was performed with Superscript IV reverse transcriptase and specific primers (5′-CCGTCAATTCMTTTGAGT-′3) as recommended by the manufacturer (Thermo Fisher Scientific, Schwerte, Germany). Residual RNA was removed by adding 1 µl RNase H (New England Biolabs, Frankfurt am Main, Germany) and incubation for 20 min at 37 °C. The V3-V4 region of the 16S rRNA gene was amplified as described previously (2) using the primers S-D-Bact-0341-b-S-17 (3) and S-D-Bact-0785-a-A-21 (4). Amplicons were purified by using MagSi-NGS PREP Plus magnetic beads as recommended by the manufacturer (Steinbrenner Laborsysteme GmbH, Wiesenbach, Germany). Library preparation and paired-end sequencing on Illumina MiSeq (2 × 300 bp mode) was done as described previously (2). We obtained 3,600,522 DNA-based and 4,321,748 RNA-based 16S rRNA amplicon sequences with 2,646,601 and 2,580,599 remaining sequences after quality filtering (Table 1). Default parameters were used for all software unless otherwise specified. Sequences were quality-filtered with fastp v0.20.0 (5) and merged with PEAR v0.9.11 (6), primer sequences clipped with cutadapt v2.5 (7). Generation of ASVs (amplicon sequence variants) by size filtering (minimum length 300 bp), dereplication, denoising, and removal of low-abundant sequences (<8 reads) and chimera removal (*de novo* and against SILVA database) was done with vsearch v2.7.1 (8). Taxonomic identity of ASVs was determined by BLASTN v2.9.0 (9) against the SILVA SSU 138 NR database (10) as described previously (2). Data analysis was performed in R v4.0.3 (11) and RStudio v1.3.1093 (12) with the ampvis2 package (13). The most abundant orders at DNA level were *Ktedonobacterales* and subgroup 2, while *Acidobacteriales* (*Acidobacteriota*) *showed* higher abundances at RNA level (Fig. 1A). Diversity increased from rainforest to managed land use systems at entire and active community level (Fig. 1B).

## Data Availability

Paired-end sequences are available at the National Center for Biotechnology Information under the Bioproject accession number PRJNA687358. Paired-end sequences are available at the National Center for Biotechnology Information under the Bioproject accession number PRJNA687358. Biosample accession numbers are as follows: BF1a (SRS8047154), BF1b (SRS8047155), BF1c (SRS8047166), BF2a (SRS8047177), BF2b (SRS8047188), BF2c (SRS8047194), BF3a (SRS8047195), BF3b (SRS8047196), BF3c (SRS8047197), BF4a (SRS8047198), BF4b (SRS8047156), BF4c (SRS8047157), BJ2a (SRS8047158), BJ2b (SRS8047159), BJ2c (SRS8047160), BJ3a (SRS8047161), BJ3b (SRS8047162), BJ3c (SRS8047163), BJ4a (SRS8047164), BJ4b (SRS8047165), BJ4c (SRS8047167), BJ5a (SRS8047168), BJ5b (SRS8047169), BJ5c (SRS8047170), BO2a (SRS8047171), BO2b (SRS8047172), BO2c (SRS8047173), BO3a (SRS8047174), BO3b (SRS8047175), BO3c (SRS8047176), BO4a (SRS8047178), BO4b (SRS8047179), BO4c (SRS8047180), BO5a (SRS8047181), BO5b (SRS8047182), BO5c (SRS8047183), BR1a (SRS8047184), BR1b (SRS8047185), BR1c (SRS8047186), BR2a (SRS8047187), BR2b (SRS8047189), BR2c (SRS8047190), BR3a (SRS8047191), BR3b (SRS8047192), BR3c (SRS8047193), BR4a (SRS8047151), BR4b (SRS8047152), and BR4c (SRS8047153).
